# A new lexicon in the age of microbiome research

**DOI:** 10.1098/rstb.2023.0060

**Published:** 2024-05-06

**Authors:** Thomas C. G. Bosch, Martin J. Blaser, Edward Ruby, Margaret McFall-Ngai

**Affiliations:** ^1^ Zoological Institute, University of Kiel, 24118 Kiel, Germany; ^2^ Center for Advanced Biotechnology and Medicine, Rutgers University, Piscataway, NJ 08854, USA; ^3^ California Institute of Technology, Pasadena, CA 91125, USA

**Keywords:** symbiosis, antimicrobial peptides, pathogen, immunity, microbe-associated molecular patterns, commensal

## Abstract

At a rapid pace, biologists are learning the many ways in which resident microbes influence, and sometimes even control, their hosts to shape both health and disease. Understanding the biochemistry behind these interactions promises to reveal completely novel and targeted ways of counteracting disease processes. However, in our protocols and publications, we continue to describe these new results using a language that originated in a completely different context. This language developed when microbial interactions with hosts were perceived to be primarily pathogenic, as threats that had to be vanquished. Biomedicine had one dominating thought: winning this war against microorganisms. Today, we know that beyond their defensive roles, host tissues, especially epithelia, are vital to ensuring association with the normal microbiota, the communities of microbes that persistently live with the host. Thus, we need to adopt a language that better encompasses the newly appreciated importance of host-microbiota associations. We also need a language that frames the onset and progression of pathogenic conditions within the context of the normal microbiota. Such a reimagined lexicon should make it clear, from the very nature of its words, that microorganisms are primarily vital to our health, and only more rarely the cause of disease.

This article is part of the theme issue ‘Sculpting the microbiome: how host factors determine and respond to microbial colonization’.

## 1. The rationale: why our language needs to be reconsidered: ‘*Nomen est omen*’ (*The name is the sign*)

While the biological concept of ‘symbiosis’ dates back hundreds of years, the word was not introduced until the late nineteenth century, when the German mycologist Anton de Bary provided the clear definition of symbiosis as the persistent living together of unrelated organisms [1]. The community of biologists studying these relationships developed a strong and lasting lexicon, that commonly defined three types of symbiosis: mutualism, commensalism, and parasitism (or pathogenesis). These subcategories were based on the effect of the association on the genetic fitness of the partners. In a mutualistic relationship, fitness of both partners is enhanced; in a commensal relationship, one partner benefits and the other is unaffected; and in a pathogenic association, one partner benefits and the fitness of the other is compromised.

Throughout the twentieth century, the groups studying these types of relationships were in different scientific cultures. Mutualistic symbioses were the purview of basic biology, where the practitioners principally studied tight relationships between a host and one or a few species of microbial partners (e.g. nitrogen-fixing rhizobia and plants, microbes and insects or marine animals that live in nutrient-poor environments, luminous bacteria and fishes or cephalopod hosts). Pathogenesis, also typically the study of two organisms, i.e. a host and a pathogenic symbiont, was principally studied in biomedicine and agriculture. Commensalism was typically used broadly in biomedicine, where it was thought that the associating microbiota neither harmed nor benefited the human host. Technological advances over the past 30 years have revealed a whole new set of dimensions to the field of symbiosis.

This Perspective is not meant to be a complete overview of our language around symbiosis, but rather it aims to clarify meanings of current usage and make specific suggestions [2] (table 1). Names give identity, provide orientation and convey meaning.

**Table 1.** Proposed changes to terms used to describe host–microbe interactions.

previous/misused term	definition	new term	definition
AMP (antimicrobial peptide/protein)	host peptide/protein that kills or inhibits pathogens	microbiota-regulating peptide/protein (MRP)	host peptide/protein that regulates the normal microbiota and/or invaders
commensal microbiota	microbes whose presence has no effect on the host	normal microbiota	typically present microbes, with or without an identified cost or benefit to the host
infection	pathogen invasion and growth in a host	colonization	microbial growth in or on a host whose effects are context dependent
pathogen-associated molecular patterns (PAMPs)	microbial products indicating the presence of a pathogen	microbe-associated molecular patterns (MAMPs)	conserved microbial products whose effects on the host are context-dependent
pathogen	disease-causing microbe, sometimes invading the normal microbiota	amphibiont	microbe with the potential of a pathogenic or beneficial effect, depending on the context
virulence factor	property of microbe that results in tissue injury or disease	colonization factor	property of microbe that promotes growth on or in host tissue, resulting in disease, beneficial or neutral effects

Here we ask: how might a change in our terms make our language more unified across the field of host–microbe interactions? Because of the current challenges to the health of all corners of the biosphere and, because microbes are foundational to all of life, we contend that it is critical to develop a common lexicon that more cogently serves the discipline of biology.

## 2. The rapid emergence of microbiome research revolutionizes the field of symbiosis

From the time of Louis Pasteur, and indeed Anton van Leuwenhoek, medical science was aware of what was called a ‘normal flora’. While studies of vertebrates revealed enormous numbers of microbial cells, a deep understanding of their diversity was hindered by the inability to culture most of them in the laboratory. A more complete awareness emerged in 1977 when Carl Woese pioneered phylogenetic taxonomy [3], i.e. a phylogeny based upon the comparison of gene sequences that encode ribosomal RNA. Those data revealed an unexpectedly vast diversity in the microbial world, and drastically changed our view of the phylogenetic relationships within the biosphere [4]. This conceptual advance was followed by development of rapid and inexpensive next-generation sequencing techniques, commercially available beginning in 2005, which led to an explosion in the discovery and characterization of a vast array of new microbial taxa [5–7].

The breakthrough of these sequencing methods revealed to the broad community of biologists a world they could not have known before. We now recognize that dynamic host–microbe interactions have shaped the evolution of life, and that all animals and plants are associated with a ubiquitous microbial world, diverse in its specific compositions [8]. Further, there is a growing recognition that most, if not all, animals and plants naturally maintain microbial communities that contribute to host health. Where such associations occur, the partners comprise a collective entity known as the ‘holobiont’ or ‘metaorganism’ [9]. In these alliances, the host depends upon its symbiotic bacteria for proper health and development, from nutrient metabolism to regulating whole life cycles [10]. These microbial partners in turn benefit from the habitats and resources that the host provides [11,12]. Indeed, since the Woesian revolution, we have discovered that:

(i) the biosphere exists as nested ecosystems, where microbes live in association with animals and plants, either as environmental cohabitants or as holobionts; in turn, the organisms making up these symbioses occur in ecological populations and communities;
(ii) in many cases, hosts and their resident microbes live in tight relationships that have shaped the partners' form and functions over evolutionary time;
(iii) the number of microbial cells that reside on and in plants and animals, including humans, can rival or exceed that of host cells [13]; and
(iv) microbes play a critical role in the regulation of the host's interface with both the biotic and abiotic environments, including the host immune and nervous systems.

This new conceptual framework has driven scientists to rethink the roles of microorganisms [14,15], and has inspired us to also rethink the language we use to describe host–microbe interactions.

## 3. Notable instances where the lexicon needs reconsideration

### (a) Host molecules

We discuss first the necessity of rethinking the lexicon with some very specific, prominent examples, beginning with a consideration of antimicrobial peptides and proteins [16], both of which have been abbreviated as ‘AMPs’ [17]. However, new findings suggest that the name no longer fits the breadth of function of these host products (table 1). Here, we argue that, when present at biologically relevant levels, such peptides and proteins actually can play both positive and negative microbiota-regulating roles, shaping the composition of the microbial community by differentially affecting its membership (table 1).

When two cationic microbiota-regulating peptides (MRPs) were identified in the haemolymph of silk moths in the early 1980s [18], they were considered to be the primary mode of insect defence against invading pathogens. The subsequent discovery of magainins in *Xenopus* frogs [19] changed the view of this class of natural antibiotics, revealing that (i) MRPs are major components of the innate immune system produced by virtually every living organism, including vertebrates [20], and (ii) they are a first line of defence against invading pathogens [21,22]. From the beginning of animal (and plant) evolution, MRPs have served a crucial role in regulating the composition of the microbiome [23]. We illustrate this phenomenon with three examples across the tree of life:

(i) in the cnidarian *Hydra*, microbial establishment in early embryos is made possible by maternally encoded antimicrobial peptides [24]. After mid-blastula transition, zygotically expressed antimicrobial peptides exert control over the microbiome. After hatching, a stable and specific microbiome is established within three to four weeks. This population shift goes hand in hand with a dramatic change in the nature of the MRPs produced, i.e. replacement of the maternally produced MRPs [24]. In adult *Hydra*, specific MRPs contribute to the host control of bacterial colonization. For example, the distribution of the cationic neuron-derived MRP 1 (NDA-1) regulates the spatial distribution of the Gram-negative bacterium *Curvibacter*, the main colonizer along the *Hydra* body column ([25] see also [26]);
(ii) recent work in *Drosophila* uncovered the selective pressures driving the evolution of MRPs and how they control constituents of the fly microbiome [16,27] (see also [28] in this issue). By screening *Drosophila* mutants lacking specific MR gene families, a surprising specificity of single peptides for single microbes was found, suggesting an origin from longstanding evolutionary relationships that select host peptides for control of environmental microbes (see also [28]). The enormous diversity of MRPs produced by *Drosophila* can be explained by the adaptation of host genetics to the microbiome. By offering insights into how host immune systems adapt to the suite of microbes associated with a particular ecological niche, the *Drosophila* studies therefore also contribute to the need to rethink the basis for the development of immune systems, across the biosphere (see also [28]); and
(iii) MRPs also are fundamental components of mammalian innate immunity, where they are often referred to as ‘defensins’. In the gastrointestinal tract, home to great numbers of symbiotic bacteria, these peptides both prevent microbes from penetrating the epithelial barrier and help shape the composition of the microbiome. As in *Drosophila,* genetic analyses can be applied in humans to learn about the role of MRPs. Inactivating mutations in the cystic fibrosis transmembrane conductance regulator cause malfunction of MRPs in response to pH changes [29]. One consequence of such mutations is that residential bacteria such as *Staphylococcus aureus* expand to great numbers, creating dense biofilms. Analysis of common diseases such as cystic fibrosis, atopic dermatitis, urinary tract infection and periodontal disease have implicated impaired expression of MRPs as contributing to the underlying pathophysiology [30–33]. These pathological conditions illustrate the importance of MRPs in regulating populations of residential organisms in addition to their roles in controlling introduced pathogens, such as *Pseudomonas* or *Stenotrophomonas* species.

Microbiota-regulating proteins are another class of molecules with which the host can sense and respond to microorganisms. Because of their deep ancestral relationships with microbes, plants and animals have evolved highly conserved proteins (e.g. Toll-like receptors) to recognize and deal with the presence of bacteria in their midst. A vast array of proteins including the interleukins, cytokines, pattern-recognition receptors, antibodies and the complement cascade are signature elements of the immune systems of animals. Recent research has clearly shown that these proteins play critical beneficial roles in the dynamics of the microbiome, which means that we must recast our view of these molecules. General overviews illustrate examples of this phenomenon in mammalian systems [34], and in invertebrates [35]. While many of the protein-family members do not have names that reflect their role in interacting with the resident microbiome, we include these MR proteins in our discussion here to underscore the importance of their more recently recognized functions in responding to the beneficial microbiota.

An illustration of the magnitude of the new findings can be found by an analysis of the literature databases. For example, a mid-2023 search in Europe PMC for publications on the topic of ‘immune proteins microbiome’ showed over 42 000 references, with greater than 99.9% appearing since 2007, and greater than 50% published in the last 3 years. Such research in taxa across the animal kingdom has revealed that immune proteins interact with the microbiome to control a variety of critical activities as disparate as: (i) circadian rhythms [17,34,36–39]; (ii) immune modulation [40–43]; and (iii) host development [44–47].

These findings make clear that MRPs and proteins do much more than just kill pathogens. They play a ‘silent’ role in human health by permitting the host to have a coordinated coexistence with environmental and symbiotic microbes, shaping the microbiome according to specific susceptibility to particular ‘antimicrobial’ molecules, and contributing to the spatial organization of the microbiota. Thus, these molecules set the prerequisites for healthy colonization of the epithelium and for the development of colonization resistance. As such, instead of ‘anti’-microbial, depending on the context, these factors could also be called ‘pro’-microbial peptides or proteins. For this reason, we believe that using the more neutral terms, MRPs and proteins, more accurately encompasses the context-dependent nature of their influence.

### (b) Microbial molecules

Microbe-associated molecular patterns (MAMPs), formerly called pathogen-associated molecular patterns (PAMPs) [46], are common cellular features specific to bacteria that are recognized by host animals and plants. Some of these elements, notably the derivatives of lipopolysaccharide on the surface of Gram-negative bacteria, and the peptidoglycan of the bacterial cell wall, have been given names directly associated with pathogenesis. Specifically, lipopolysaccharide (LPS) is often called ‘endotoxin’, and the monomer of peptidoglycan has been referred to as a ‘cytotoxin’ because, in experimental pathogenesis models, these molecules can be highly perturbing to host cells and, thus, were considered archetypal ‘virulence factors’. Then, in 2004, the LPS and peptidoglycan of *Vibrio fischeri* were shown to synergistically induce normal development of host tissues upon symbiont colonization, a finding that prompted the authors to propose the use of a new acronym, ‘MAMPs’, instead of PAMPs [48]. Now widely used, the term MAMPs also recognizes that these molecules are, in fact, characteristic of the envelopes of myriad species of bacteria, only a few of which cause pathogenesis. In fact, the ‘cytotoxic’ peptidoglycan derivative that was discovered to induce host-tissue development in the squid-vibrio association was later found to also be required to drive the normal maturation of gut-associated lymphoid tissue in mammals [45]. MAMPs have now been found to be critical cues in several other symbiotic associations of both plants [49] and animals [50].

## 4. Rethinking immunity

We propose that, in the study of host–microbe interactions, some rethinking and clarification of the concept of immunity is also necessary. With the discovery of infectious diseases, immunity was initially conceived as the host's response to invading pathogens [51]. However, as discussed above, essentially all of an animal or plant's microbiome is not pathogenic, but is instead composed of many beneficial and essential symbionts, contributing to a variety of functions that the host has outsourced to them, including promoting resistance to invaders. Thus, the traditional view of the ‘protective’ role of the immune system does not adequately account for its importance to a host's normal physiology. The immune system does not only fend off pathogens; for example, innate immune systems evolved to manage and exploit beneficial microbes [52]. As first proposed [53], and later embraced [40], a memory-based (i.e. adaptive) immune system may have evolved in vertebrates because of the need to recognize and manage the complex communities of beneficial microbes that are in fact a hallmark of vertebrate life. These roles in shaping and maintaining the community ecology of the microbiota shifts the perception of the primary activity of an adaptive immune system from one of defending against an occasional pathogen to one of continually monitoring and promoting a myriad of beneficial relationships. The implications of such a revised concept of immunity have broad significance for both life scientists and philosophers considering immunology, ecology and the cognitive sciences [54].

## 5. The launch of a new lexicon

### (a) The term ‘pathogen’

The word ‘pathogen’ is a trigger word, burdened with concepts that spend most of their time in consideration of harmful infections. Despite the popular use of the generic term ‘germs’, scientists now widely recognize that it is incorrect to consider human-linked microbes as primarily producing disease; the vast majority of host–microbe interactions are either beneficial or benign [55]. In fact, most animals and plants generally have only a handful of associated microbial species that are ‘frank pathogens’ [56], as well as others that are typically part of the normal microbiota but, under certain circumstances, can become harmful: the so-called ‘opportunistic pathogens' [57]. In phylogenetic trees, many pathogens belong to genera whose species are predominantly members of the normal microbiota (e.g. *Neisseria*, *Clostridium*, *Bacteroides, Campylobacter*). Nevertheless, because of our historical interest in the diseases of mankind, for several prominent genera, the first species to be described was one that caused a human disease (e.g*. Neisseria meningitidis*). Only later, and often aided by subsequent molecular descriptions, were other, closely related host-associated species described that were non-pathogenic, or even beneficial. Thus, the biological ancestry of many if not most of these lineages appears not to have arisen as pathogens, particularly in the case of human-disease associated species [58]; that is, pathogenesis is a derived character. Finally, it is important to recognize that specific environmental conditions may be necessary for the appearance of virulence in host-associated bacteria; that is, their pathogenicity is almost always context-dependent [59,60]. For example, even among closely related animals (e.g. mice and humans) a deadly pathogen in one, can be a benign symbiont in the other [61].

In the 1960s, Theodore Rosebury, a microbial ecologist, introduced the concept of ‘amphibiosis’, which refers to biological relationships between two organisms that may lead to damage or benefit, depending on context [62]. A classical amphibiont of humans is *Helicobactor pylori*, which leads to increased risk of conditions such as peptic ulcer disease and adenocarcinoma of the distal stomach, but decreases the risk of more proximal gastric and esophageal adenocarcinomas [63], and conditions involving other organs, such as childhood onset asthma [64,65] (see below). The more closely we examine the biology of ‘pathogens’ that are typically part of the microbiota, such as *Clostridium difficile* and viridens streptococci, the more we find their other, beneficial, properties. In this context, it is worth remembering that *C. difficile* was first isolated from stool of a healthy newborn [66], and is nearly universally found in healthy children in the first year of life. To make the ambiguity of the term ‘pathogen’ clear, Mazmanian has proposed the term ‘pathobiont’ [67], which has the same meaning as Rosebury's earlier term.

### (b) Miscellaneous issues with the lexicon: toxins, virulence factors and commensals

There are many other terms in the old lexicon that either quickly imply an exclusively negative function or are misleading. An example is the word ‘toxin’. In bivalve molluscs, cholera toxin is much more than a toxic protein; instead, under natural conditions it may be involved in other, perhaps beneficial, interactions with soluble haemolymph factors and the signalling pathways of the haemocyte [68].

Similarly, in *H. pylori*, a protein called CagA was discovered [69,70] that was injected directly into the gastric epithelium through a type-IV secretion system [71,72], continuously over the decades of gastric colonization. Originally described as a ‘virulence factor’ whose presence increased risk of both peptic ulcer disease and gastric cancer, it also may be considered as a ‘protective factor’ because its presence is associated with protection against oesophageal adenocarcinoma and childhood onset asthma [54,73,74], with support from experimental studies in mice [75]. For the amphibiotic *H. pylori,* the CagA protein is best considered as a ‘host-interaction factor’ (HIF), intensifying the cellular interactions of the host with this luminal organism, with both pathogenic and beneficial functions, depending on context. The toxins of *C. difficile* might be similarly considered as HIFs, intensifying immune adjuvancy in the young, and colitis in the old [76].

Another frequently misused term is ‘commensal’. As reported previously [77,78], the term ‘commensal’ was adopted by nineteenth-century physicians to describe what were then believed to be the ‘harmless’ and thus inconsequential bacteria digesting and growing on the food present in the gut of humans. From that viewpoint, their choice of the term ‘commensal’ was entirely consistent with its accepted definition (of benefit to one partner, and neutral to the other). However, we now recognize that such bacteria are not neutral in their effects on the fitness of their hosts; in fact, a vast literature has arisen over recent decades whose major conclusions can be summarized as: most of the microbes associated with the biosphere, from the soil to the human gut, are essential for keeping life processes balanced, and keeping their specific animal and plant hosts healthy [79]. Thus, it is unfortunate that the very literature providing convincing evidence of microbiota benefits continues to refer to these organisms as ‘commensals’. This archaic misuse of the term commensal not only perpetuates imprecision in the lexicon, but also creates an unnecessary quandary: what do we call those microbial species that, in fact, have no significant impact on the host's fitness? The answer is simple; such organisms, should they exist, are commensals, while those that provide demonstrable benefit are beneficial. However, our present knowledge of absence of benefits (or costs) may be overturned by future discoveries. Thus, it might be more accurate to simply refer to such relationships in the most broad sense, as mutualism, as described above.

## 6. Further measures must follow

The new language must also be reflected in teaching. When microbes are discussed in the classroom or lecture hall, they are too often labeled as ‘germs’, ‘bad’, ‘dangerous’, ‘pathogenic’ or ‘harmful’; this biases our approaches in the clinic as well [80]. Are we therefore adequately explaining our findings and knowledge to the public? We believe we can do better, and we offer a framework (table 1) to consider new terms adapted to our better understanding of the relationships between microbes and their hosts. However, certain parts of the lexicon serve us well. A good example is the ‘pattern-recognition receptors' or PRRs [81]. That phrasing holds no specific judgment on fitness cost or benefit to the host or symbiont. This quality holds true for several types of PRRs, including Toll-like receptors and nucleotide-binding oligomerization domain-like receptors.

In summary, the shift in our understanding of host–microbe interactions in recent decades requires a rethinking of our lexicon. To stay consistent with new research findings, it is time to abandon once and for all the ‘war metaphor’ [82] and its accompanying language used to describe host–microbe interactions. When we begin to use and further develop this new lexicon, we will be better positioned to accurately describe the complex interactions between hosts and their microbes for what they really are: an essential basis of life.

## Data Availability

This article has no additional data.
